# Comparison of invasive blood pressure monitoring vs. non-invasive blood pressure monitoring in critically ill children receiving vasoactive agents—a prospective observational study

**DOI:** 10.3389/fped.2024.1376327

**Published:** 2024-06-03

**Authors:** Sachin Shah, Amita Kaul, Rohini Nagarkar, Amol Thorat

**Affiliations:** ^1^ Department of Neonatal and Pediatric Intensive Care Services, Surya Mother and Child Superspecialty Hospital, Pune, India; ^2^ Department of Pediatrics, Surya Mother and Child Superspecialty Hospital, Pune, India

**Keywords:** non-invasive blood pressure (NIBP), invasive blood pressure (IBP), vasoactive agents, shock, paediatric intensive care unit (PICU)

## Abstract

**Objective:**

The primary aim of this study was to compare non-invasive blood pressure (NIBP) measurement using the automated oscillometric method with invasive blood pressure (IBP) measurement using peripheral arterial line insertion in critically ill children receiving vasoactive agents.

**Design:**

Single-centre, prospective cohort study.

**Setting:**

Tertiary care 15 bedded Pediatric ICU in Urban Indian city.

**Subjects:**

All critically ill children between the ages of 1 month to 16 years with shock on vasoactive medications and with IBP monitoring.

**Results:**

Forty children with 1,072 paired BP measurements were incorporated in the final analysis. Among all normotensive children (Total number of paired measurements = 623) receiving vasoactive agents, Bland–Altman analysis revealed an acceptable agreement between Invasive mean blood pressure (MBP) and non-invasive MBP with a bias of −2.10 mmHg (SD 11.35). The 95% limits of agreement were from −24.34 to 20.14 mmHg. In children with hypotension (Total number of paired measurements = 449), Bland–Altman analysis showed disagreement between Invasive MBP and non-invasive MBP i.e., a bias of −8.44 mmHg (SD 9.62). The 95% limits of agreement were from −27.29 to 10.41 mmHg.

**Conclusion:**

A limited agreement exists between invasive blood pressure (IBP) and non-invasive blood pressure (NIBP) measurements in critically ill children requiring vasoactive agents. This discrepancy can lead to either an underestimation or an overestimation of blood pressure. While NIBP can serve as a screening tool for hemodynamically stable children, those who are hemodynamically unstable and necessitate the initiation of vasoactive agents should undergo IBP monitoring.

## Introduction

Precise measurement of blood pressure (BP) holds significant importance when caring for critically ill children. The decision-making process for initiating, adjusting, or discontinuing vasoactive medications relies on various clinical parameters, with blood pressure being a particularly crucial determinant.

BP is measured in Pediatric Intensive Care Unit (PICU) commonly by two methods namely non-invasive measurements with a cuff by oscillometric technique (NIBP) or invasive blood pressure measurements (IBP) through an arterial catheter. Peripheral arterial canulas (PAC) are utilized for IBP monitoring and although errors related to movement artifacts and calibration of an arterial line are possible, IBP monitoring is regarded as the gold standard. However, PACs are not risk-free, and complications such as thrombosis, ischemia, local skin discoloration, and gangrene have been cited (1). Also, sometimes it may be hard to secure arterial access or the PAC may have to be taken out due to adverse effects. In such a scenario, clinicians have to rely on NIBP measurement. BP cuffs of different sizes are currently available for all pediatric age groups. The oscillometric method is most commonly used as it is user friendly.

Most of the studies do not show clear agreement or disagreement between NIBP and IBP in critically ill children admitted to PICU (1–3). Nevertheless, none of the studies conducted thus far have specifically analyzed the concordance between NIBP and IBP in critically ill children who require vasoactive agents.

Establishing a satisfactory agreement between NIBP and IBP can make it possible to manage critically ill children with shock using NIBP measurements. This can have important implications in a resource-limited setting.

The primary aim of this study was to compare NIBP measurement using automated oscillometric method with IBP measurement utilizing peripheral arterial cannulation in critically ill children receiving vasoactive agents.

## Material and methods

The study was conducted in a tertiary care 15-bed PICU in urban India from 1st November 2021 till 30th November 2022. Informed written consent was taken from the parents. All critically ill children with IBP monitoring and receiving vasoactive agents between the ages of 1 month to 16 years were prospectively enrolled in the study. The study was approved by the institutional ethics committee (SRERC Protocol Submission no: SRERC/2021/TH/01 dated 04.02.2021 title: *Comparison of Invasive blood pressure monitoring* vs. *non-invasive blood pressure monitoring in critically ill children receiving vasoactive agents—a prospective observational study”)* was registered with the clinical trials registry of India (number CTRI/2021/10/037569). All the research procedures were per the ethical standards of the Surya Mother and Child Care Scientific Research and Ethical Review Committee and with the amended Helsinki Declaration of 1975.

### Inclusion criteria

All critically ill children with shock needing vasoactive medications and have IBP monitoring.

### Exclusion criteria

Children with contraindications to IBP monitoring such as injuries,burns or gangrene of extremities.

**The indication for PAC** was the need for vasoactive agents.

The radial artery was the preferred site for PAC. After cannulation of the artery, the line was connected to an extension tubing and transducer (Edward Lifesciences services GmbH, Germany) and Drager Vista 120 model multipara monitor (Draeger, Luebeck, Germany) for continuous blood pressure monitoring. The arterial waveform was continuously displayed on the monitor. The monitors underwent a biannual examination, and calibration was conducted annually by a biomedical engineer by the standards established by the National Accreditation Board for Hospitals.

Only in-house PICU consultants on call or the PICU fellow were privileged to put arterial lines. No medications, electrolytes, additional fluids, or blood products were administered through the line, it was used only to collect blood for blood gas analysis and routine laboratory studies.

The NIBP measurements were taken by the nurse attending to the child by using Drager Vista 120 model multipara monitor (Draeger Medical GMBH, Luebeck, Germany). The midarm circumference was measured and then the cuff size (NIBP cuff size, Draeger, Luebeck, Germany) was selected as per the manufacturers' recommendation. The width of the bladder of the cuff covered approximately 50% of the midarm circumference. NIBP measurements were obtained in the same limb as the peripheral arterial line.

In hypotensive children, NIBP and IBP measurements were documented before initiation of vasoactive agents and subsequently after each dosage adjustment, till the target BP was attained. Children on vasoactive agents and with stable BP underwent hourly recordings of IBP and NIBP. Initially, IBP readings were documented and later NIBP measurements were taken within one minute using the same monitor. These were termed paired measurements as they were taken within 1 minute interval of each other. Only a single NIBP measurement was taken to minimize discomfort. All measurements were preferably taken in a quiet resting state. Sedation or analgesia was not used in non-ventilated children, while it was used as per need utilizing the Richmond Agitation- Sedation scale in ventilated children (4).

The target invasive mean Blood pressure (mmHg) was ≥5th centile for age (defined as 40 + 1.5 × age in years). The vasoactive agents were titrated to this operational definition. Hypotension was defined as invasive mean blood pressure <5th centile for age (5).

A fixed protocol of starting vasoactive agents was followed and all patients with hypotension were started on epinephrine in a starting dose of 0.05 µg/kg/min and the dose was escalated every 15 min if the target blood pressure was not reached. If a patient was present in a pulse-less state, then the starting dose of epinephrine was 0.1 µg/kg/min. All patients initially were started on vasoactive agents through external jugular venous cannulation if available or a wide-bore peripheral cannula. Within an hour of starting vasoactive agents, a central venous catheter was inserted and vasoactive agents were administered through the same. After reaching a dose of 0.3 µg/kg/min of epinephrine if the target blood pressure was not reached, we added norepinephrine. The starting dose of norepinephrine was 0.05 µg/kg/min. If the need for norepinephrine exceeded 0.3 µg/kg/min, vasopressin was introduced. Hydrocortisone was added when the norepinephrine and epinephrine were at a dose of ≥0.3 µg/kg/min. Vasopressin was commenced at a dose of 0.0002 IU/kg/min. Milrinone was used in patients who had achieved normal blood pressure after initial vasopressors but still had low mixed venous saturation or abnormal perfusion (CFT > 2 s) in a starting dose of 0.33 µg/kg/min. The highest vasoactive inotrope score was noted at the time of data entry.

Our predefined criteria for an acceptable NIBP value was a difference of 5 mmHg or less when compared to IBP (6).

Prospective data collection was conducted until either the PAC was *insitu* or vasoactive agents were ceased. The information was systematically recorded using a predefined form and then entered into MS Excel software.

### Primary outcome measure

Level of agreement between IBP and NIBP measurements.

The following comparisons were performed:

1. Normotensive readings in children receiving vasoactive agents
2. Hypotensive readings in children receiving vasoactive agents

### Secondary outcome measures

Complications associated with PAC including thrombosis, ischemia, infection, bleeding, etc.

### Sample size calculation

Differences in mean BP exceeding 5 mmHg are clinically significant in critically ill children and differences surpassing 10 mmHg are deemed as clinically unacceptable (6). To detect a difference of 5 mmHg between IBP and NIBP with a SD of difference of 5 mmHg, assuming an alpha of 0.05 and beta of 0.2 (power of 90%), using a two-sided paired *t*-test, we calculated a sample size of 32 patients. Assuming a 20% dropout rate, we chose a sample size of 40 patients.

## Statistical analysis

Statistical analysis was conducted utilizing IBM SPSS 23 version (NY, USA: IBM corp). The agreement between IBP and NIBP was evaluated using Bland- Altman analysis. We calculated agreement (bias, mean difference) and precision (1.96 SD of the difference) corresponding to the 95% limits of agreement. This provides an interval within which 95% of the differences between measurements by the two methods are expected to lie.

## Results

**Figure 1:**
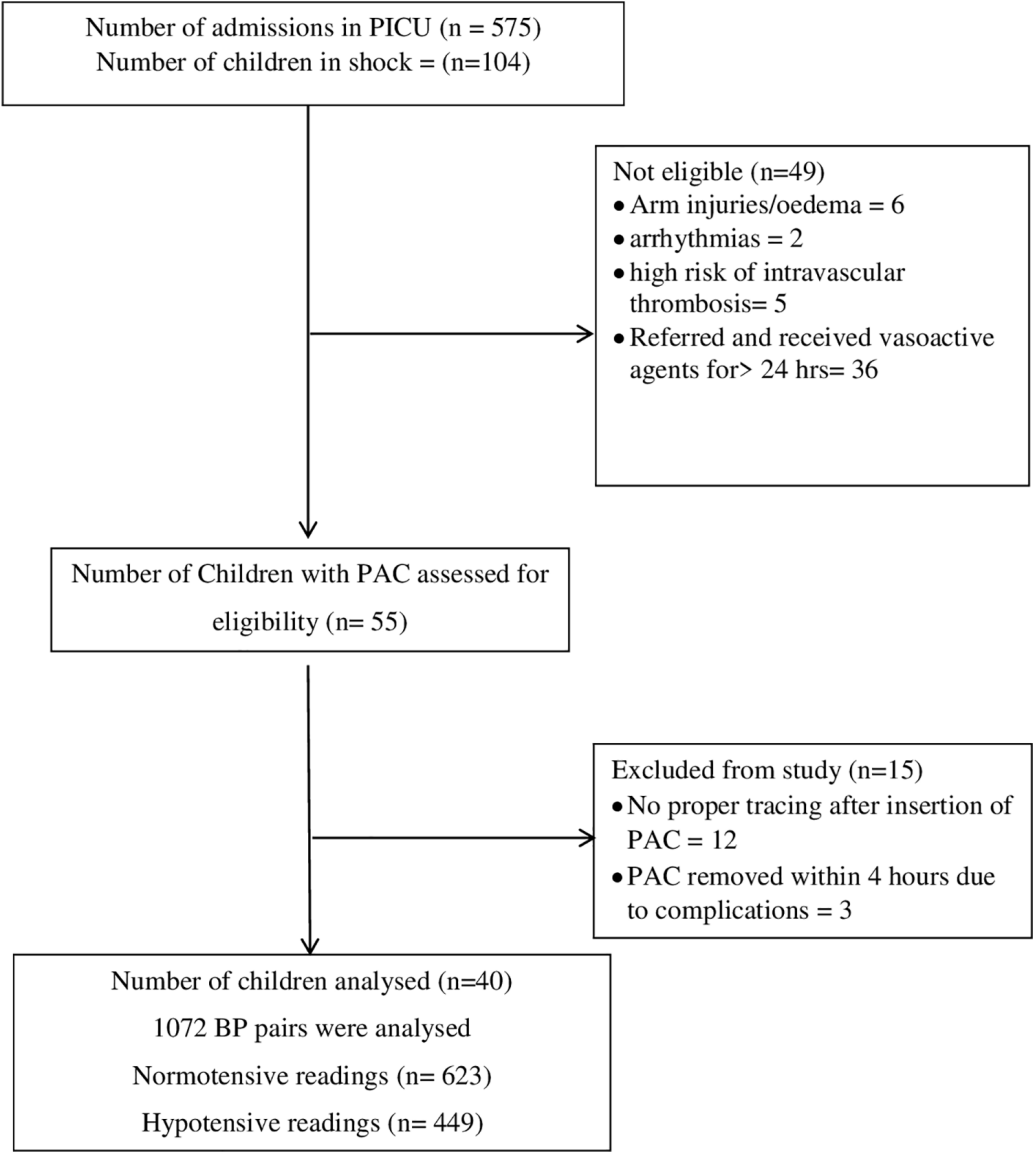
Study flow chart, (PICU, paediatric intensive care unit; PAC, peripheral arterial line).

During the study period, 575 children were admitted to PICU. Out of 575 children admitted, 104 children had features suggestive of shock. Of these forty children were eligible for the study (Details in the study flow chart) (Figure 1). Clinical characteristics of enrolled children have been tabulated (Table 1). The Median (IQR) age of enrolled children was 5 (4, 8) years. The male-to-female ratio was almost 1:1. The right radial artery was cannulated in a majority of the cases [34(85%)]. Sepsis and Multisystemic Inflammatory Syndrome-Children were the most common causes of shock. Median (IQR) Vasoactive-Inotropic score (VIS) and Paediatric Index of Mortality (PIM2) score were [34.5 (20, 58.6) and 2.25 (−4.5, 13.6)].

**Table 1:** Clinical characteristics of enrolled children.

Total number of children studied (*n* = 40)	
Age in years (median, IQR)	4 (5, 8)
Male [*n* (%)]	12 (52.2)
Weight in kilograms (Median, IQR)	20 (12, 28)
Right Radial arterial cannulation [*n* (%)]	34 (85%)
Left Radial arterial cannulation [*n* (%)]	6 (15%)
Total number of paired measurements (*n*)	1,072
Duration of a peripheral arterial line, (mean, SD), hours	30.16 ± 16.05
Vasoactive-Inotropic Score (VIS) (Median, IQR)	34.5 (20, 58.6)
Pediatric index of mortality (PIM) 2 Score (Median, IQR)	2.25(−4.5, 13.6)
Diagnosis	
Septic shock	18 (45%)
Multisystemic inflammatory syndrome –children	8 (20%)
Dengue shock	12 (30%)
Meningoencephalitis	1 (2.5%)
Others	1 (2.5%)
Reason for removal of PAC (*n*, %)	
Elective removal due to lack of need	19 (47.5%)
Does not bleed back or dampening of tracing	16 (40%)
Non elective removal due to complication	5 (12.5%)
Complications (*n*, %)	5 (12.5%)
Blanching of skin	2
Local swelling	1
Leakage or bleeding around insertion site	2
Accidental dislodgement	0
Discolouration of digits	0
Local infection	0

**Figure 2:**
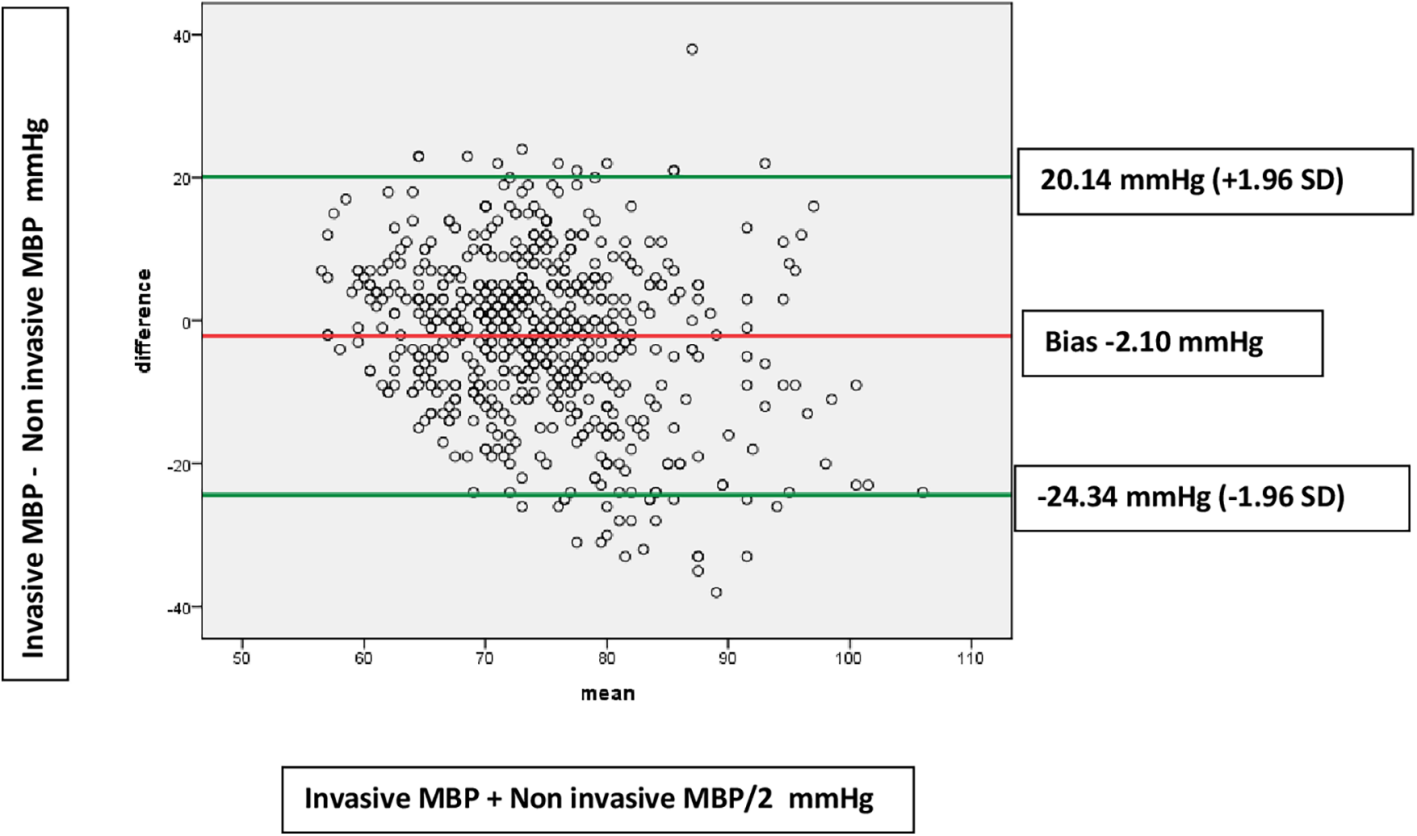
Bland-Altman plots showing agreements between invasive MBP and non-invasive MBP measurements in normotensive patients (623 paired measurements). Bias: −2.10 (SD 11.35). Limits of agreement −24.34 to 20.14 mmHg.

**Figure 3:**
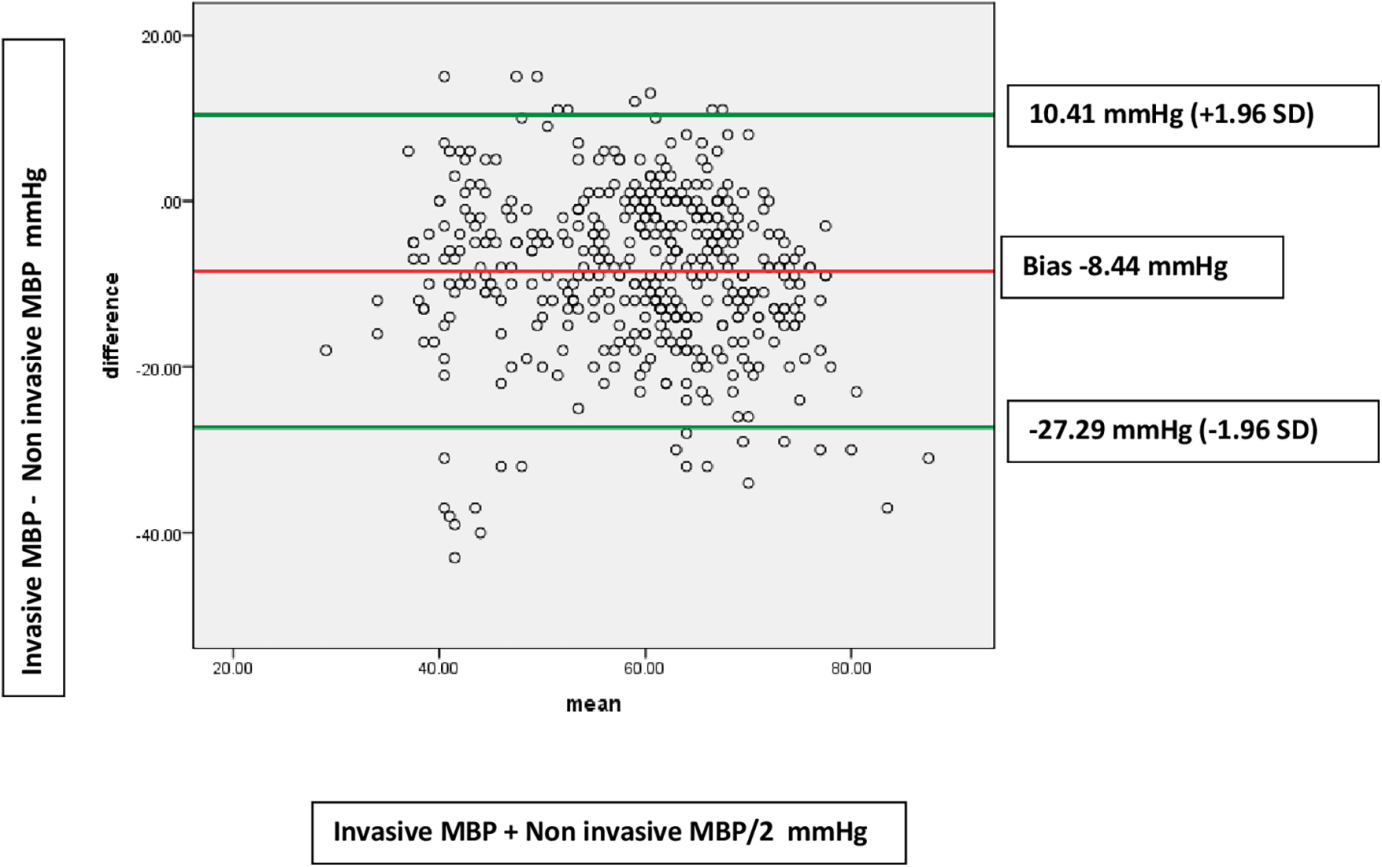
Bland-Altman plots showing disagreements between invasive MBP and non-invasive MBP measurements in hypotensive patients (449 paired measurements). Bias: −8.44 (9.62). Limits of agreement −27.29 to 10.41 mmHg.

Among all normotensive children (Total number of paired measurements = 623) receiving vasoactive agents, Bland–Altman analysis revealed an acceptable agreement between Invasive mean blood pressure (MBP) and non-invasive mean blood pressure MBP with a bias of −2.10 mmHg (SD 11.35) (Figure 2). The 95% limits of agreement were from −24.34 to 20.14 mmHg. Also, for normotensive children receiving vasoactive agents, Bland–Altman analysis for agreement between Invasive systolic blood pressure (SBP) and non-invasive SBP showed an acceptable bias of −2.52 mmHg (SD 15.08). The 95% limits of agreement were from −32.07 to 27.03 mmHg. On the other hand, in children with hypotension (Total number of paired measurements = 449), Bland–Altman analysis showed disagreement between Invasive MBP and non-invasive MBP i.e., a bias of −8.44 mmHg (SD 9.62). The 95% limits of agreement were from −27.29 to 10.41 mmHg (Figure 3). The Bland Altman analysis on enrolled children in both normotensive as well as hypotensive group is shown in Table 2.

**Table 2:** Bland Altman analysis on enrolled children.

		Bias (SD) in mmHg	95% limits of agreement (mmHg)	Number of paired measurements	Invasive BP in mmHg (median, IQR)	Non invasive BP in mmHg (median, IQR)
Normotensive readings in children receiving vasoactive agents	Invasive MBP vs. noninvasive MBP	−2.1 (11.35)	−24.34 to 20.14	623	62 (57–69)	64 (58–71)
	Invasive SBP vs. noninvasive SBP	−2.52 (15.08)	−32.07 to 27.03	623	94 (86–102)	95 (88–103)
Hypotensive readings in children receiving vasoactive agents	Invasive MBP vs. non invasive MBP	−8.44 (9.62)	−27.29 to 10.41	449	52 (43–59)	59 (51–66)
	Invasive SBP vs. noninvasive SBP	−12.38 (14.86)	−41.50 to 16.74	449	80 (65–90)	91 (77–98)

## Discussion

In our study, we observed a substantial variation in the 95% limits of agreement between IBP and NIBP resulting in potential overestimation or underestimation of blood pressure. But certain interesting observations were noted. In the subgroup of children who were normotensive but receiving vasoactive agents, the bias between invasive blood pressure (systolic and mean) and non-invasive systolic blood pressure (systolic and mean) was within our pre-decided acceptable limits of 5 mm Hg. Hypotensive children receiving vasoactive agents exhibited a bias of more than 5 mmHg between invasive and non-invasive blood pressure readings. The limits of the agreement were also wide. The bias was more pronounced for systolic BP measurements as compared to mean BP measurements.

In PICU, where critically sick children are being managed, blood pressure remains one of the important parameters to decide to start the vasoactive agents and titrate them to achieve the endpoints. Certainly, blood pressure (BP) assessment needs to be considered alongside other parameters, including heart rate, capillary refill time, functional echocardiography, etc.

Relying excessively on NIBP for decisions regarding vasoactive drug management may pose challenges due to its weak correlation with IBP.

The bias that has come up as a result of our study could be due to erroneous NIBP readings or erroneous IBP readings. While considered the gold standard, there is a possibility that IBP measurements may not always be fully reliable. Moreover, the use of a small-diameter catheter in narrow vessels may result in inaccurate systolic blood pressure readings (7). Technical factors such as the presence of air bubbles, zeroing, and damping can contribute to inaccuracies in IBP measurements. The nurses were taught to monitor and troubleshoot for air bubbles, damping, and kinking while handling PAC. Minor kinks underneath the tape may go unnoticed. Such errors are unlikely to be common and introduce a systematic bias in the findings. Thus, a comparison of MAP was conducted across various subgroups, given that it remains unaffected by damping. The NIBP measurements were obtained in a state of rest and quiet, interpretation of this state can vary among the nurses. A single measurement was taken to avoid discomfort to the sick child. Studies have shown that repeating up to 3 measurements improves the accuracy with the 2nd or 3rd reading being more accurate (8).

We ensured that the correct size NIBP cuff was used after measurement of midarm circumference. These NIBP cuffs have a range of around 4 cm. The cuff bladder may cover more than 50% of mid-arm circumference at the lower range of mid-arm circumference inspite of being technically correct for the child. However, it is practically impossible to have one NIBP cuff matching to a single mid-arm circumference.

The oscillometric technique for NIBP relies on the concept that pulsatile blood flow induces oscillations in the arterial wall which are then transmitted to the cuff. The point of maximum oscillations corresponds to MAP while SBP and DBP are derived from a predetermined algorithm that varies among devices. Given its higher accuracy compared to the other two measures, it is more rational to compare MAP using both IBP and NIBP. Our study found a lower bias between invasive MAP and non-invasive MAP compared to systolic pressures.

Joffe et al. evaluated the use of invasive and non-invasive blood pressure in 100 critically ill children (147 arterial lines and readings) and found poor agreement between invasive and non-invasive BP measurements (2). The majority of the cohort was post-cardiovascular patients, while sepsis was the major diagnosis in our cohort. Krishna et al. compared invasive blood pressure vs. non-invasive blood pressure by oscillometric method in 50 critically ill children (100 readings) and noted that NIBP underestimates systolic BP and overestimates diastolic BP (9). The bias was less for mean BP but still not clinically acceptable. Additionally, the number of readings was much smaller in both studies.

The strengths of our study include a large number of readings in a homogenous cohort of children with shock and on vasoactive agents rendering it the most extensive study, to the best of our knowledge, in examining such a cohort. Secondly, NIBP obtained in our study was taken from the same limb as that of IBP thereby eliminating the potential bias of measuring BP along different arterial vasculature.

One potential limitation is that this was conducted at a single center, and there is a possibility that despite the application of standard precautions, the NIBP and IBP measurements could be vulnerable to ordinary human errors. There might be factors beyond the device and patient that could influence the level of agreement which we were unable to assess. However, our pragmatic approach mirrors the routine clinical situation in any PICU.

We acknowledge that it is neither feasible nor practical to perform IBP monitoring in all sick children and we need to rely on NIBP measurements. In situations where there are concerns about the hemodynamic status and NIBP values, it is advisable to prioritize IBP monitoring. The decision to initiate and titrate vasoactive agents should be made accordingly.

Furthermore, it is advisable to depend on repeated IBP/NIBP measurements in conjunction with other clinical findings before initiating vasoactive agents. We should rely more on MBP as the primary value since it is the sole measured value in the NIBP technique and is also subject to less variation in the IBP technique. In the future, emerging techniques in NIBP measurement, incorporating machine learning, pulse waveform contour analysis, and algorithm-based blood pressure sensors, could hold promise and enhance accuracy (9, 10).

An apprehension regarding the utilization of PAC is the potential occurrence of complications. However, the occurrence of major complications in our study was infrequent. PACs were electively removed in 47.5% of children while in 40%, the lines had to be removed due to damping. The most frequent complications seen with PAC were inability of the line to bleed back and trace dampening. No untoward effects were observed following the removal of these lines. Severe complications like discolouration of digits, blanching of the skin, or bleeding occurred in 5 (12.5%) children.Blanching and discoloration resolved following the removal of the arterial line and warming of the contralateral limb, while the bleeding ceased after the application of local pressure. No long-term consequences were noted till discharge from the hospital. Similar rates of complications have been reported by other studies (11, 12).

## Conclusions

In critically ill children requiring vasoactive agents, there is a limited level of agreement between IBP and NIBP measurements, resulting in potential overestimation or underestimation of blood pressure. The observed bias was lower for mean blood pressure measurements compared to systolic BP measurements and for normotensive cases compared to hypotensive cases. While NIBP may function as a screening method for hemodynamically stable children, those who are hemodynamically unstable and require initiation of vasoactive agents should undergo IBP monitoring. Newer non-invasive methods should be explored where securing a PAC may not be feasible to monitor and manage children receiving vasoactive agents.

## Data Availability

The datasets used and or analysed during the study are available from the corresponding author on reasonable request.
